# Combination of Cisplatin, Ifosfamide, and Adriamycin as Neoadjuvant Chemotherapy for Extremity Soft Tissue Sarcoma: A Report of Twenty-Eight Patients

**DOI:** 10.1097/MD.0000000000002611

**Published:** 2016-01-29

**Authors:** Bing Wang, Xiuchun Yu, Songfeng Xu, Ming Xu

**Affiliations:** From the Department of Orthopedic, the General Hospital of Jinan Military Commanding Region. Jinan, China.

## Abstract

To investigate the clinical efficacy of neoadjuvant chemotherapy in the treatment of extremity soft tissue sarcomas (STSs).

We retrospectively analyzed 28 patients with extremity STS that received 2 cycles of preoperative and 6 cycles of postoperative neoadjuvant chemotherapy between May 2009 and June 2012. Chemotherapy comprised intravenous cisplatin (DDP) (120 mg/m^2^, for 1 day), followed 1 week later with 5 days 2 g/m^2^ ifosfamide (IFO) and 3 days 30 mg/m^2^ adriamycin (ADM). CT scans of the lungs and X-ray films of the lesion sites were reviewed.

Eighteen patients were treated for primary tumor and 10 for tumor recurrence. Overall tumor diameter ranged from 8 to 30 cm based on body surface measurement. A total of 224 cycles of chemotherapy were carried out and patients were followed up for 12 to 59 months. Twenty-five patients underwent wide resection surgery (89.2%), and 3 underwent amputation (10.7%). Disease-free survival was realized in 20 patients and 3 patients survived with tumors. Two-year disease-free survival rate was 71.4%, and overall 2-year survival rate was 82.1%. Postoperative metastases were observed in 5 patients, and all died of lung metastases. Postoperative recurrence was observed in 4 patients (including 1 patient occurred metastases later). Tumor size was reduced by 30% ± 11.3% on average after the preoperative chemotherapy, and was reduced by 43% ± 7.8% in 22 patients with tumors >15 cm in the diameter. Twelve patients achieved partial remission, 14 stable disease and 2 experienced progressive disease. Objective response rate was 42.9%. Disease control rate was 92.9%. Chemotherapy was well tolerated in all the patients. Main adverse reactions were transient and resolved after chemotherapy.

Neoadjuvant chemotherapy is effective in the treatment of extremity STS.

## Abstract 2

Supplemental Digital Content is available in the text

## INTRODUCTION

Soft tissue sarcomas (STSs) originate from embryonic mesoderm mesenchymal tissues. STS mainly presents as a relatively slow growing malignant tumor that lacks a complete capsule, and growth is invasive. While STSs account for less than 1% of all cancers in the United States, the incidence rate of STSs is approximately 2 per 100,000 population. STSs can occur at any age and in any part of the body.

At present, first-line treatment for STS involves radical surgery or radiotherapy, but treatment efficacy is limited and in most patients death occurs due to distal metastasis. The 5-year overall survival rate for STS is approximately 50% to 60%.^1–3^ Roughly 10% of patients already have distal metastasis at diagnosis, and 80% of patients develop metastasis within 2 to 3 years.^4^

Treatment of STS typically involves a combination of chemotherapy and radical surgery. Since 1993, our department has managed STS with surgery, radiotherapy, chemotherapy, and other comprehensive methods involving malignant soft tissue tumor limb salvage, and this treatment regimen has improved survival rate, rate of limb salvage, and quality of life.^5^ In a portion of patients with STS after surgery, chemotherapy may be continued to reduce incidence of local recurrence, but micrometastases or systemic metastasis are the real threat to the lives of patients. Systemic chemotherapy can effectively combat distant metastasis in patients with advanced STS,^6^ but for the early primary STS patients, the value of chemotherapy remains controversial.

The clinical validity of chemotherapy in STS, especially neoadjuvant chemotherapy, has not yet been clinically confirmed. In order to evaluate the value of neoadjuvant chemotherapy in this clinical setting, in this report we have summarized our experience with application of neoadjuvant chemotherapy in the treatment of 28 patients with extremity STS.

## MATERIALS AND METHODS

### Patients

We retrospectively reviewed the medical records of 28 patients, treated for STS with neoadjuvant chemotherapy at the Orthopedic Department of the General Hospital of Jinan Military Commanding Region, between May 2009 and June 2012.

The study was approved by the Ethics Committee of General Hospital of Jinan Military Commanding Region. Written informed were waived because of the retrospective nature of this study.

### Clinical Care

Location, size, and scope of tumor was assessed using lesion enhanced computed tomography (CT) and/or magnetic resonance imaging (MRI). Primary tumors were determined to be STS by tissue biopsy. Tumor recurrence was confirmed with primary pathological section after consultation with a pathologist, the criteria were referred to the previous reference.^3^

Patients received preoperative chemotherapy composed of intravenous cisplatin (DDP) (120 mg/m^2^, for 1 day), followed 1 week later with ifosfamide (IFO) (2 g/m^2^ for 5 days) and adriamycin (ADM) (30 mg/m^2^, for 3 days) (DIA scheme).

During chemotherapy, antiemetic drugs and liver detoxification drugs (3.6 g glutathione, delivered intravenously) were administered where required. Mesna was administered to protect the bladder and kidneys before and after administration of IFO. When white blood cell count fell below 3.5 × 10^9^/L, granulocyte colony-stimulating factor was administered.

Chemotherapy efficacy was assessed by CT and/or MRI after 2 cycles, then after 2 further weeks, the patients underwent surgery. Tumor was resected in 25 patients, and limb was amputated in 3 patients. Stitches were removed after 2 weeks, and the 3rd cycle of chemotherapy was applied. Three of the 5 patients that experienced metastasis and 4 with recurrent disease, received postoperative 3-dimensional conformal radiotherapy, at a dose ranging from 40 to 60 Gy, as recommended.^7,8^

**FIGURE 1:**
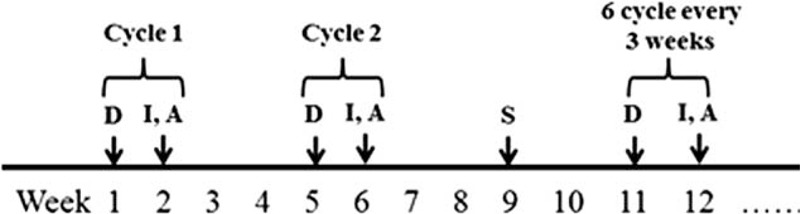
DIA chemotherapy, A = adriamycin, D = cisplatin, I = ifosfamide, S = surgery.

After surgery, a total of 6 cycles of DIA chemotherapy were administered at 3 week intervals (Figure 1), however in the last 3 cycles, ADM was not administered and only DDP and IFO were administered. Holter monitoring was applied during the last 3 cycles to assess cardiac activity, but no cardiac complications were observed.

Patients were followed up monthly for 2 years after surgery. Lung lesions were monitored by MRI, CT, ultrasound, and X-ray every 3 months. At each follow-up, the presence of recurrence, metastases, and the time of death was recorded.

### Evaluation of Chemotherapy Toxicity

The American National Cancer Institute Common Terminology Criteria for evaluation standard of Adverse Events (Version 4)^9^ was used to evaluate the main adverse reactions during chemotherapy. Neutropenia was estimated by routine blood testing and treated with granulocyte stimulating factor when it occurred. In this study, the myelosuppression was not severe. We tested for anemia, cardiotoxicity, and renal impairment during chemotherapy, but these adverse effects were not observed in this group.

Adverse events were divided into 5 stages: grades 1 , mild, no or minimal symptoms, no treatment; grades 2, moderate, smaller, local or noninvasive treatment, causes mild or limited activities of daily living; grades 3, severe but not immediately life-threatening, leading to hospitalization or prolonged hospitalization or disability, daily life was limited; grades 4, life-threatening requiring emergency treatment; and grades 5, associated with adverse events leading to death.

### Evaluation of Chemotherapy

Changes in lesions on imaging before and after chemotherapy were based on the European Cancer Conference, the response evaluation criteria in solid tumors Response Evaluation Criteria In the Solid Tumour (RECIST 1.1).^10^ When evaluating chemotherapy, the response was classified as: complete remission (CR), partial remission (PR), stable (SD), and progressive disease (PD). The CR refers to all target lesions that disappeared completely; PR refers to the longest diameter of target lesion from the radiographic measurement that reduced more than 30%; PD refers to the longest diameter of target lesions that increased more than 20%, or the number of minimum diameter increased more than 5 mm, or the appearance of new lesions; SD refers to the longest diameter has reduced less than PR or increased less than PD. The CR + PR were summed to give the objective response rate, and CR + PR + SD were summed for the disease control rate.

### Statistical Methods

Patient data were presented as means ± standard deviation and percentage, and statistical analyses were calculated using SPSS19.0 (SPSS Inc, Chicago,IL), and the 2-year disease-free survival rate and overall survival rate were calculated by the Kaplan–Meier method.

## RESULTS

### Patient Demographic and Clinical Characteristics

The sample included 18 males and 10 females, with a mean age of 39.5 years (18–62 years; median age 35 years). Eighteen patients were treated for primary tumor and 10 for tumor recurrence. Tumor types included 12 patients with malignant fibrous histiocytoma, 4 patients with synovial sarcoma, 3 patients with leiomyosarcoma, 4 patients with rhabdomyosarcoma, 2 patients with epithelioid sarcoma, and 3 patients with Ewing sarcoma (soft tissue). The tumor was located in the femur in 8 patients, the hip in 4 patients, the shoulder in 4 patients, axillary in 2 patients, popliteal fossa in 2 patients, calf in 5 patients, and forearm in 3 patients. Overall tumor diameter ranged from 8 to 30 cm (mean diameter 15 cm) based on body surface measurement (Table 1). Patients received 38 weeks of DIA chemotherapy, including 2 cycles of preoperative chemotherapy and 6 cycles of postoperative chemotherapy. Twenty-five patients underwent wide resection surgery, and 3 underwent amputation. The limb salvage rate was 89.2%, and amputation rate was 10.7%. All patients completed chemotherapy, for a total of 224 chemotherapy cycles.

**TABLE 1:**
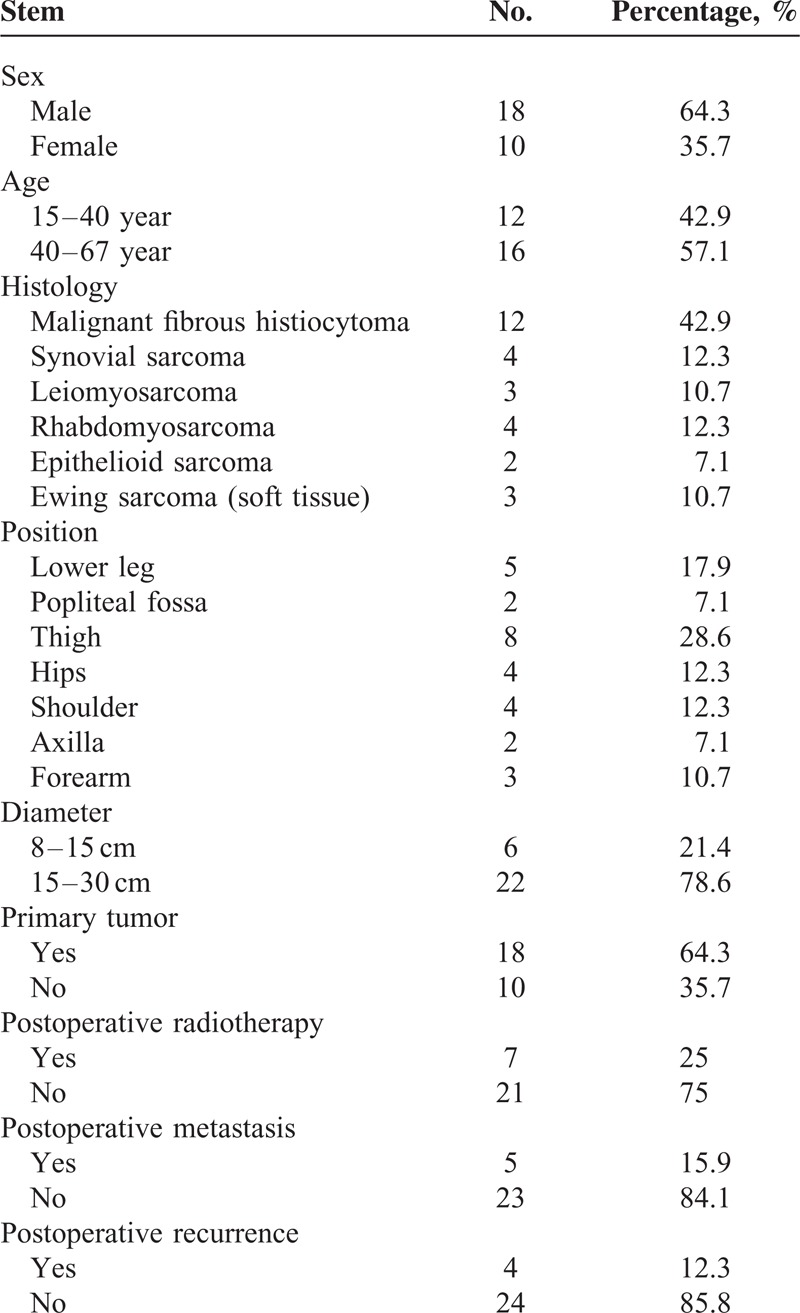
The Baseline Characteristic of the 28 Patients

### Clinical Outcome

**FIGURE 2:**
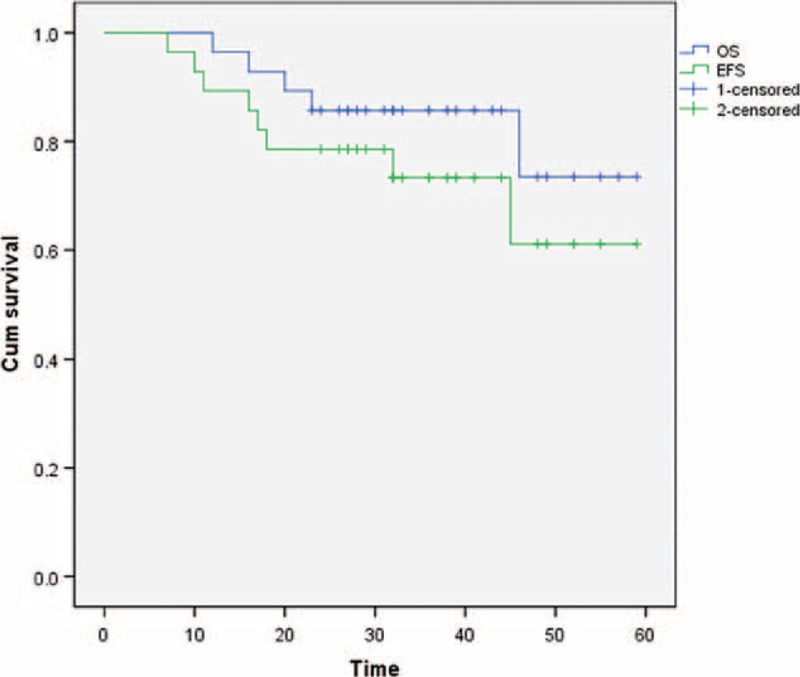
The 2 year overall survival rate 82.1%, the 2 year disease-free survival rate 71.4%.

All surgeries were successful, and complete wound healing was achieved as scheduled in all patients. The mean total blood loss was 260 ± 27.9 mL (range: 50–600 mL). Median duration of follow-up for the 23 patients was 32 months (range between 12 and 59 months). In total, 5 patients died and 23 survived. Disease-free survival was achieved in 20 patients and 3 patients survived with tumors. Tumor recurrence occurred in 4 patients. One patient developed epithelioid sarcoma and died as a result of lung metastasis. Two patients who developed malignant fibrous tissue cell tumor, and 1 patient who developed leiomyosarcoma underwent surgical extended resection, with adjuvant radiotherapy. Five patients developed pulmonary metastasis, 2 cases of epithelioid sarcoma, and 3 cases of malignant fibrous histiocytoma (Table 1). The 2-year OS rate was 82.1%, and the 2-year DFS rate was 71.4% (Figure 2).

### Chemotherapy Toxicity

Chemotherapy was well tolerated and there was high compliance with treatment. In total, 224 cycles of chemotherapy were administered. The main adverse events included bone marrow suppression, nausea and vomiting, abnormal liver function, and hair loss (Table 2). These symptoms were transient and resolved after chemotherapy was discontinued.

**TABLE 2:**
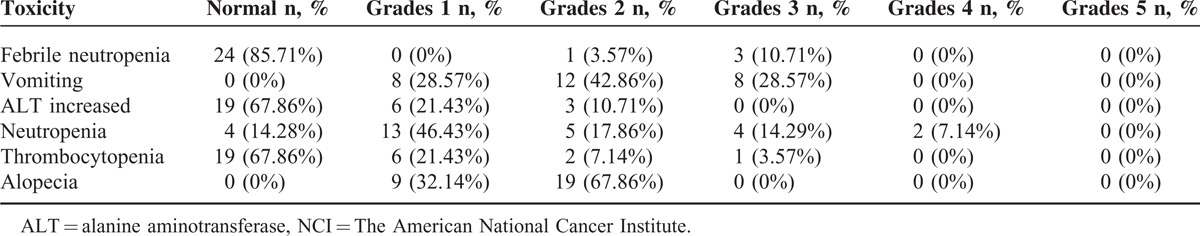
NCI Common Terminology Criteria for Adverse Events (Version 4)

### Evaluation of Chemotherapy

In this patient group, tumor size was reduced by an average of 30% ± 11.3% after preoperative chemotherapy. In the 22 patients with tumors with a diameter over 15 cm at initiation, tumor diameter was reduced by an average of 43% ± 7.8%. According to RECIST 1.1 criteria, 0 patients achieved CR, 12 patients achieved PR, 14 patients achieved SD, and 2 patients developed PD. The objective response rate was 42.9%, and disease control rate was 92.9%.

## DISCUSSION

Early clinical studies suggest that chemotherapy did not improve OS or relapse rate in patients with STS.^11,12^ However, the relevance of these findings is limited by the complex range of STS pathologies, the variable sensitivity to chemotherapeutic drugs, and single or combined application of drugs. A meta-analysis by Pervaiz et al^13^ demonstrated that chemotherapy can reduce the local recurrence rate, distant metastasis rate, and increase OS. The Radiation Therapy Oncology Group initiated phase II trial 9514 and showed a significant reduction in distant metastases, with a highly significant gain in DFS and OS after an aggressive neoadjuvant chemotherapy.^14,15^ This regimen also led to long-term survival benefits.^16^ Recently the NCCN soft tissue tumor and treatment guidelines (Online: http://www.nccn.org/professionals/physician_gls/pdf/sarcoma.pdf) have emphasized the importance of adjuvant and neoadjuvant chemotherapy before surgery. Although there are no large randomized trials, preoperative chemotherapy can reduce the extent of surgical resection, reduce the impact on limb function, particularly for high grade sarcoma, and a treatment option has been reported to improve the local control rate, OS rate, and DFS rate.

Neoadjuvant chemotherapy can be used as treatment in high risk STS patients, including those with high tumor grade (G2-G3), and for deep or particularly large tumors.^17,18^ This is especially the case for the primitive neuroectodermal tumor, and rhabdomyosarcoma where neoadjuvant chemotherapy has become the treatment of choice.^19,20^ Gortzak et al^21^ reported that adjuvant chemotherapy in high risk patients had little effect on OS, but reduced local recurrence and distant metastasis.

In theory, neoadjuvant chemotherapy has advantages over postoperative adjuvant chemotherapy.^18,22,23^ In our study, first preoperative chemotherapy can establish sensitivity to chemotherapy, and the regimen can be modified where ineffective. Second preoperative chemotherapy can control establishment of micrometastasis, and third preoperative chemotherapy can significantly reduce tumor volume, and surrounding soft tissue edema, and is conducive to the smooth removal of limb STS.

**FIGURE 3:**
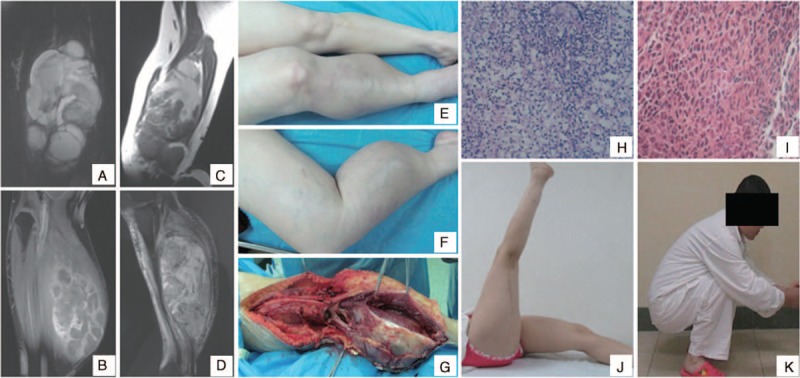
(A,B) Magnetic resonance imaging (MRI) before chemotherapy, the large soft tissue tumor in right lower limb, from posterior thigh to posterior lower leg, its boundary was not clear. (C,D) MRI after 2 cycles of chemotherapy, the tumor volume reduced, and its boundary was clear. (E,F) Appearance before operation. The tumor was large, and the en-bloc resection was difficult. (G) There was clear boundary of the tumor in operation, and its membrane integrity. (H) Biopsy pathology: small cell malignant tumor, primitive neuroectodermal tumor (HE × 200). (I) Postoperative pathology: the tumor cells exhibited necrosis (HE × 200). (J,K) Postoperative appearance after 3 years. The right lower limb function is good, and the patient can squat, jump, and run.

In this group of patients, after preoperative chemotherapy, the tumor volume decreased by 30%. In some patients, it was difficult to completely remove the large STS, but with preoperative chemotherapy to shrink the tumor volume, the patients who required amputation underwent treatment for limb salvage. This group of primitive neuroectodermal tumor patients included 1 patient with preoperative right lower limb large soft tissue mass formation, from the right thigh rear extending into the right posterior leg. The tumor volume was huge and the boundary was not clear. Preoperative biopsy revealed a primitive neuroectodermal tumor, and simple surgical resection was difficult. After 2 cycles of DIA chemotherapy the tumor volume was reduced, and lower limb function was retained after extensive tumor resection. The patient's gait was roughly normal, and the patient can squat, jump, and run (Figure 3).

Chemotherapy drugs currently used in the treatment of STS, include ADM and IFO. Single drug chemotherapy of STS achieved an OS of 14% to 30%.^24^ Large doses of ADM + IFO (AI) increases the effectiveness of chemotherapy to 35% to 45%.^25^ A study of 134 patients with giant limb and pelvic malignant STS used neoadjuvant chemotherapy including 2 cycles of preoperative of 120 mg/m^2^ DDP + 60 mg/m^2^ ADM and 4 cycles this chemotherapy regimen postoperatively with 2 cycles of high-dose (14 g/m^2^) IFO. Over an average of 5 years of follow-up, the DFS rate reached 80%, and the OS rate was 88%.^26^ Another regimen of IMAP (IFO, mitomycin, ADM, and DDP) plus granulocyte-macrophage colony-stimulating factor followed by preoperative irradiation and subsequent limb-sparing surgery is satisfactory as initial treatment for primary extremity STSs.^27^ In our study, the combined use of DDP + IFO + ADM can effectively control tumor cell proliferation. The results confirmed the efficacy of this regimen in the treatment of extremity STS, with good patient tolerance, and high compliance.

However, the conclusions of this study are restricted by the limitations of its small sample size and relatively short follow-up period. We also did not take the pathological type into account, which may have directly influenced drug sensitivity of sarcoma (Supplementary material).

## CONCLUSION

In summary, preoperative chemotherapy can significantly reduce STS tumor volume, reduce the surrounding soft tissue edema, and can control micrometastases. Neoadjuvant chemotherapy can improve the OS rate and DFS rate, limb salvage treatment for STS of the limbs, with high patient satisfaction and acceptable toxicity. Neoadjuvant chemotherapy with a DIA regimen is effective in the treatment of STS.

## Supplementary Material

Supplemental Digital Content
