# Supplementary material for: Combination of Cisplatin, Ifosfamide, and Adriamycin as Neoadjuvant Chemotherapy for Extremity Soft Tissue Sarcoma: A Report of Twenty-Eight Patients
Source: Medicine (Baltimore). 2016 Jan 29;95(4):e2611. doi: 10.1097/MD.0000000000002611 (PMC5291584; doi:10.1097/MD.0000000000002611)
Supplement: Supplemental Digital Content [file medi-95-e2611-s001.doc]

**Case study**

In September 2012 a 15-year-old female patient presented with a mass on the right forearm and right hand numbness that limited daily living activities. Upon physical examination, the right forearm appeared swollen, and a 20 × 15 cm tenacious mass with unclear boundary was observed. The mass was soft, and there were no superficial varicose veins. The local skin temperature was slightly elevated, but the skin color was normal. The arm circumference that 5 cm proximal from wrist crease was 24 cm and 17 cm at right and left, respectively. Passive extension of the wrist was limited. The patients reported hypoesthesia of the palm side of fingers of the right hand, and Tinnel’s sign was positive. The right radial artery pulse was normal. MRI showed soft tissue swelling in the right forearm, patchy high signal intensity, and heterogeneous enhancement after contrast. An lesion with a clear boundary could be seen compressing the blood vessels and muscle. Pathological rhabdomyosarcoma was confirmed on biopsy. Preoperative treatment consisted of 2 cycles of the DIA regimen: DDP (120 mg/m^2^, for 1 day), 1 week after administration of IFO (2 g/m^2^ for 5 days), and ADM (30 mg/m^2^, for 3 days). Pain relief was reported after chemotherapy. The soft tissue mass was significantly reduced, the boundary was clear (approximately10 × 5 cm), and the arm circumference was17.5 cm at right and 17 cm at left.

During surgery under brachial plexus anesthesia, the right forearm soft tissue tumors were resected, tendons were reconstructed, and posterior cubital lymph node was excised. The soft tissue tumor wrapped the radial artery and radial nerve, and superficial flexor tendon of the ring finger and flexor digitorum superficialis tendon of the little finger. The tumor had not invaded the vasculature or nerves, and had a clear edge and complete capsule. Since the tumor edge was sharp, it was freed together with the flexor digitorum superficialis tendon. The tendon anastomosis was removed, and excised from the flexor digitorum profundus tendon. In the posterior elbow the medial head vein was separated from several enlarged lymph nodes with a maximum diameter of about 1 cm. A postoperative forearm plaster cast was applied to immobilize the limb for about 6 weeks. Symptomatic treatment was applied as required and the patient was instructed to exercise the upper limb function and dress the wound. Postoperative pathology confirmed rhabdomyosarcoma, but lymph nodes were metastasis free. Two weeks after surgery, the six cycles of DIA regimen chemotherapy was administered. After 2 years of follow-up, the right upper limb function has recovered well, and no recurrence or metastasis was reported (Figure S1).

Figure S1


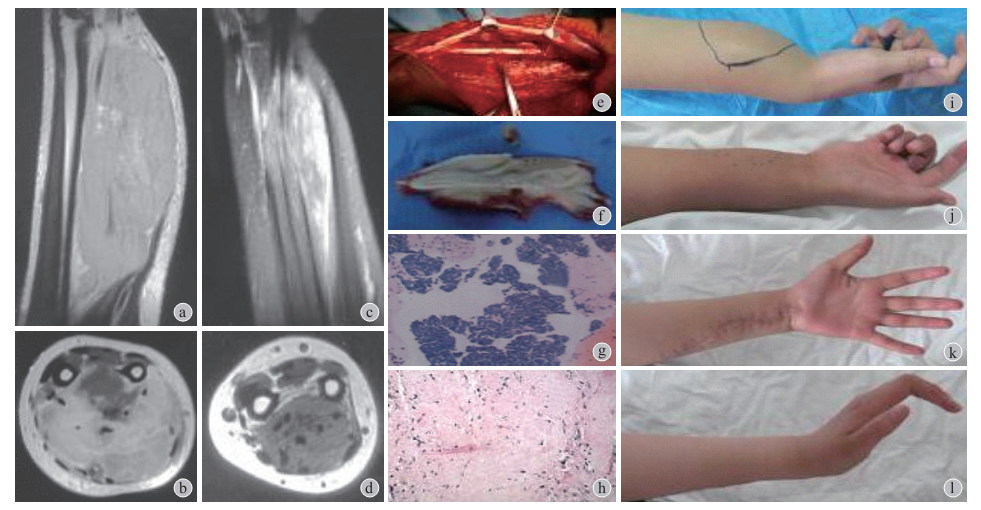


a, b, i. MRI and photo before chemotherapy. The boundry of the soft tissue tumor in the right forearm was not clear. Patchy high signal intensity was observed, and the blood vessels and surrounding muscles were stressed.

c,d, j. MRI and photo after 2 cycles of chemotherapy. The tumor was reduced by about 50% in size, and its boundary was clear.

e,f. The soft tissue tumor wrapped the superficial flexor tendon of the finger. There was one enlarged lymph node behind the elbow.

g. Biopsy pathology: malignant tumor cell nests (HE×200).

h. The postoperative pathology: Rhabdomyosarcoma, the tumor cells showed degeneration and necrosis (HE×200).

k,l. Postoperative appearance after 2 years. Joint function recovered to normal.
